# The Madagascar hissing cockroach as a novel surrogate host for *Burkholderia pseudomallei*, *B. mallei* and *B. thailandensis*

**DOI:** 10.1186/1471-2180-12-117

**Published:** 2012-06-22

**Authors:** Nathan A Fisher, Wilson J Ribot, David DeShazer

**Affiliations:** 1 Center for Genomic Sciences, United States Army Medical Research Institute of Infectious Diseases, Fort Detrick, Frederick, MD, 21702, USA; 2 Bacteriology Division, United States Army Medical Research Institute of Infectious Diseases, 1425 Porter St., Fort Detrick, Frederick, MD, (301) 21702-5011, USA; 3 University of Maryland School of Medicine, Baltimore, 21201 (WA), MD, USA

**Keywords:** Pathogenesis, Melioidosis, Glanders, Virulence, Surrogate host, Type VI secretion system

## Abstract

**Background:**

*Burkholderia pseudomallei* and *Burkholderia mallei* are gram-negative pathogens responsible for the diseases melioidosis and glanders, respectively. Both species cause disease in humans and animals and have been designated as category B select agents by the Centers for Disease Control and Prevention (CDC). *Burkholderia thailandensis* is a closely related bacterium that is generally considered avirulent for humans. While it can cause disease in rodents, the *B. thailandensis* 50% lethal dose (LD_50_) is typically ≥ 10^4^-fold higher than the *B. pseudomallei* and *B. mallei* LD_50_ in mammalian models of infection. Here we describe an alternative to mammalian hosts in the study of virulence and host-pathogen interactions of these *Burkholderia* species.

**Results:**

Madagascar hissing cockroaches (MH cockroaches) possess a number of qualities that make them desirable for use as a surrogate host, including ease of breeding, ease of handling, a competent innate immune system, and the ability to survive at 37°C. MH cockroaches were highly susceptible to infection with *B. pseudomallei*, *B. mallei* and *B. thailandensis* and the LD_50_ was <10 colony-forming units (cfu) for all three species. In comparison, the LD_50_ for *Escherichia coli* in MH cockroaches was >10^5^ cfu. *B. pseudomallei*, *B. mallei*, and *B. thailandensis* cluster 1 type VI secretion system (T6SS-1) mutants were all attenuated in MH cockroaches, which is consistent with previous virulence studies conducted in rodents. *B. pseudomallei* mutants deficient in the other five T6SS gene clusters, T6SS-2 through T6SS-6, were virulent in both MH cockroaches and hamsters. Hemocytes obtained from MH cockroaches infected with *B. pseudomallei* harbored numerous intracellular bacteria, suggesting that this facultative intracellular pathogen can survive and replicate inside of MH cockroach phagocytic cells. The hemolymph extracted from these MH cockroaches also contained multinuclear giant cells (MNGCs) with intracellular *B. pseudomallei*, which indicates that infected hemocytes can fuse while flowing through the insect’s open circulatory system in vivo.

**Conclusions:**

The results demonstrate that MH cockroaches are an attractive alternative to mammals to study host-pathogen interactions and may allow the identification of new *Burkholderia* virulence determinants. The importance of T6SS-1 as a virulence factor in MH cockroaches and rodents suggests that the primary role of this secretion system is to target evasion of the innate immune system.

## Background

*Burkholderia mallei* is an obligate parasite of horses, mules and donkeys and no other natural reservoir is known [1]. The organism is a nonmotile gram-negative bacillus that is closely related to *Burkholderia pseudomallei* and *Burkholderia thailandensis*. *B. pseudomallei* is a pathogenic microbe that causes the glanders-like disease melioidosis [2] and *B. thailandensis* is a weakly pathogenic soil saprophyte [3]. While a handful of *Burkholderia* virulence determinants have been identified using rodent models of infection [4], research on the molecular mechanism(s) of pathogenesis is still a fertile area. *B. malleiB. pseudomallei*, and *B. thailandensis* are able to survive and replicate inside phagocytic cells in a process that involves escape from the endocytic vacuole, replication in the cytosol, intra- and intercellular spread by actin polymerization, and fusion with uninfected cells to form multinucleated giant cells (MNGCs) [4]. Gram-negative pathogens often use secretion systems to deliver virulence factors to the cytosol of host cells, where they modulate cell physiology to favor the microbe. The exploitation of host phagocytic cells by *B. pseudomallei* involves two type III secretion systems (T3SS-1 & T3SS-3) [5-7], a type V secretion system (BimA) [8], and the cluster 1 type VI secretion system (T6SS-1) [9]. T6SS-1, occasionally referred to as *tss-5*[10], is also important for host cell interactions and virulence in *B. mallei* and *B. thailandensis*[11,12].

Small mammal models of infection have long been employed to characterize virulence factors of bacterial pathogens, but over the last decade there has been an increase in the use of surrogate hosts to study the pathogenic mechanisms of bacteria [13,14]. Several surrogate hosts have been used as alternatives to mammals to study virulence factors and host-pathogen interactions with *B. pseudomalleiB. mallei*, and *B. thailandensis*, including *Galleria mellonella* larvae (wax worms) [15,16], *Dictyostelium discoideum* (phagocytic amoeba) [17], *Caenorhabditis elegans* (soil nematode) [18-20], and *Solanum lycopersicum* (tomato plantlets) [21]. These alternative hosts have allowed the identification of new *Burkholderia* virulence determinants and have confirmed the importance of virulence factors previously characterized using rodent models of infection.

Insects are popular alternatives to mammalian hosts in large-scale screening studies, owing largely to the high degree of similarity between the innate immune systems of insects and mammals [22]. In both, the recognition of pathogen-associated molecular patterns (PAMPs) by Toll receptors (insects) and Toll-like receptors (mammals) results in the production of antimicrobial peptides [23]. Furthermore, insect hemocytes and mammalian neutrophils can both engulf and kill most invading microorganisms [24]. Insects are also afforded protection from microorganisms through the coagulation and melanization of hemolymph, but they do not have an adaptive immune system.

In addition to biological similarities, several logistical issues contribute to the recent adoption of insects as alternative hosts for bacterial pathogens. Insects can be readily obtained, housed, and cared for at considerable cost savings compared to mammals. Moreover, the use of insects is not governed by animal use regulations or committees and even very large-scale experiments using insects are considered ethically acceptable. As a possible insect alternative to mammalian models of infection, we tested several *B. pseudomallei, B. mallei,* and *B. thailandensis* strains against juvenile Madagascar hissing cockroaches (MH cockroaches) obtained from a commercial vendor (Carolina Biological Supply Company). MH cockroaches are readily available, easily cultured, and reproduce rapidly. They are larger than wax moth larvae, slow moving compared to other species of cockroaches, and have a substantive carapace. These characteristics make them easier to manipulate and inoculate with known numbers of bacteria compared with other species of insects commonly used for similar studies. MH cockroaches thrive at 37°C, a characteristic that is essential for the analysis of mammalian pathogens.

In this study, we found the MH cockroach to be a suitable surrogate host for *B. pseudomallei, B. mallei,* and *B. thailandensis. Burkholderia* type VI secretion system mutants were attenuated in MH cockroaches, which is consistent with what is seen in rodent models of infection [9,25]. *B. pseudomallei* multiplied inside MH cockroach hemocytes and may be the primary mechanism by which this pathogen avoids elimination by the MH cockroach innate immune system. The results suggest that MH cockroaches are a good alternative to mammals for the study of *Burkholderia* species and possibly other mammalian pathogens.

## Results and discussion

### *B. pseudomallei* is virulent in the MH cockroach and T6SS-1 mutants exhibit attenuated virulence

**Figure 1:**
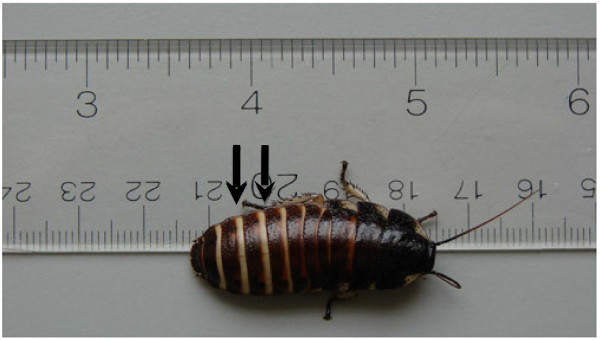
**A representative juvenile Madagascar hissing cockroach used as a surrogate host for*B. pseudomallei*,*B. mallei*, and*B. thailandensis*infection studies.** The black arrows show the locations where bacteria were inoculated into the dorsal abdominal section of the MH cockroach, between the third and the fifth terga from the posterior.

**Figure 2:**
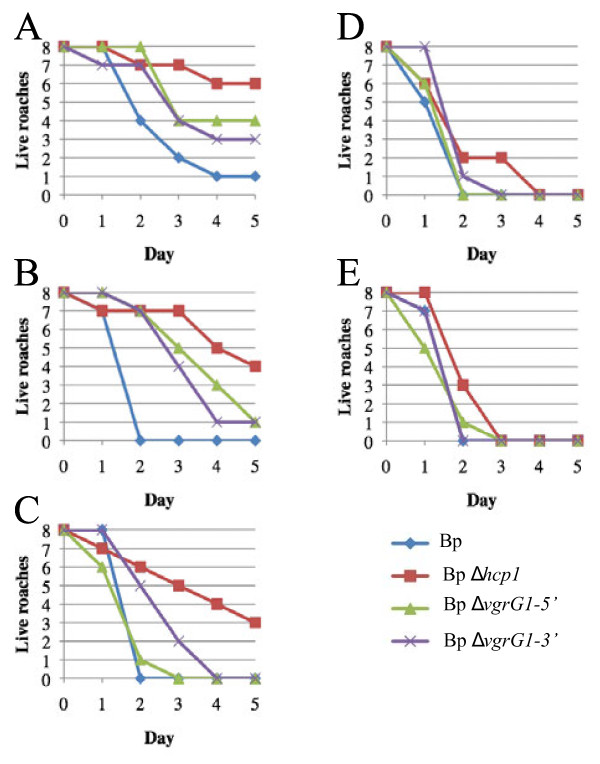
***B. pseudomallei*is virulent for the MH cockroach and T6SS-1 mutants are attenuated.** Groups of eight MH cockroaches were challenged by the intra-abdominal route of infection and MH cockroach deaths were monitored for 5 days at 37°C. (**A**) 10^1^ cfu. (**B**) 10^2^ cfu. (**C**) 10^3^ cfu. (**D**) 10^4^ cfu. (**E**) 10^5^ cfu. Bp, K96243; Bp *Δhcp1*, DDS1498A; Bp *ΔvgrG1-5’*, DDS1503-1A; Bp *ΔvgrG1-3’*, DDS1503-2A.

In an attempt to determine if the MH cockroach might serve as a surrogate host for *B. pseudomallei*, we challenged juvenile MH cockroaches (Figure 1) with K96243 and T6SS mutant derivatives. T6SS-1 is a critical virulence determinant for *B. pseudomallei* in the hamster model of infection [9], while T6SS-2, T6SS-3, T6SS-4, T6SS-5, and T6SS-6 are dispensable for virulence in hamsters. Groups of eight MH cockroaches were challenged by the intra-abdominal route with 10^1^-10^5^ bacteria and deaths were recorded for 5 days at 37°C (Figure 2).

Figure 2A shows that only one MH cockroach survived for 5 days after challenge with 10^1^*B. pseudomallei* K96243 (Bp), demonstrating that the 50% lethal dose (LD_50_) is <10 bacteria. Similarly, the LD_50_ for K96243 in the hamster model of infection was <10 bacteria [9]. *B. pseudomallei Δhcp1* is a derivative of K96243 that lacks the essential tail tube component of the T6SS-1 structural apparatus (Hcp1) and is highly attenuated in the hamster [9,26]. *B. pseudomallei Δhcp1* was also attenuated in the MH cockroach (Figure 2A-E) and the LD_50_ was ~ 2 x 10^2^ bacteria on day 5, which was >20 times higher than the K96243 LD_50_ (Table 1). In addition, a dose response was readily apparent with this strain. As the challenge dose increased from 10^1^ to 10^5^ bacteria, the number and rate of MH cockroach deaths increased accordingly (Figure 2A-E). It took a challenge dose of 10^4^*Δhcp1* to kill all eight MH cockroaches, whereas the minimum lethal dose for K96243 was only 10^2^ bacteria (Figure 2). The results demonstrate that *B. pseudomallei* is highly virulent in MH cockroaches and that T6SS-1 is a critical virulence factor in this insect host. Furthermore, there is a clear correlation between the virulence capacity of *B. pseudomallei* in the MH cockroach and the hamster (Table 1).

**Table 1:** Relative virulence of bacterial strains in Syrian hamsters and Madagascar hissing cockroaches

**Bacterial strain**	**Syrian hamster LD_50_^a^**	**Madagascar hissing cockroach LD_50_**
*E. coli*		
MC4100	ND^b^	> 10^5^
B/r	ND	>10^5^
*B. pseudomallei*		
K96243	<10	<10
DDS1498A *(Δhcp1*)	>1000	207
DDS0518A (*Δhcp2*)	<10	<10
DDS2098A *(Δhcp3*)	<10	<10
DDS0171A *(Δhcp4*)	<10	<10
DDS0099A *(Δhcp5*)	<10	<10
DDL3105A (*Δhcp6*)	<10	<10
DDS1503-1A (*ΔvgrG1-5’*)	102	<10
DDS1503-2A (*ΔvgrG1-3’*)	>450	<10
1026b	<10	<10
MSHR305	ND	<10
*B. mallei*		
SR1	<10	<10
DDA0742 *(Δhcp1*)	>10^3^	>10^3^
*B. thailandensis*		
DW503	ND	<10
DDII0868 *(Δhcp1*)	ND	>10^3^

^a^ LD_50_, 50% lethal dose [9,25,33]; ^b^ ND, not determined.

*B. pseudomallei ΔvgrG15’* and *ΔvgrG13’* are K96243 derivatives that have deletions within the gene encoding the tail spike protein (VgrG1) of the T6SS-1 structural apparatus [9,26]. These mutants were more virulent than *B. pseudomallei Δhcp1* in the hamster model of infection [9], but were less virulent than K96243 (Table 1). There was no difference in the LD_50_ of *ΔvgrG15’ΔvgrG13’*, and K96243 in MH cockroaches (Table 1), however, there was a slight but not statistically significant difference in the time to death with these strains. In general, it took longer for MH cockroaches infected with *ΔvgrG15’* and *ΔvgrG13’* to die relative to K96243 (Figure 2A-C). Thus, these strains appear to have an intermediate virulence phenotype in both MH cockroaches and in hamsters (Table 1 and Figure 2).

**Figure 3:**
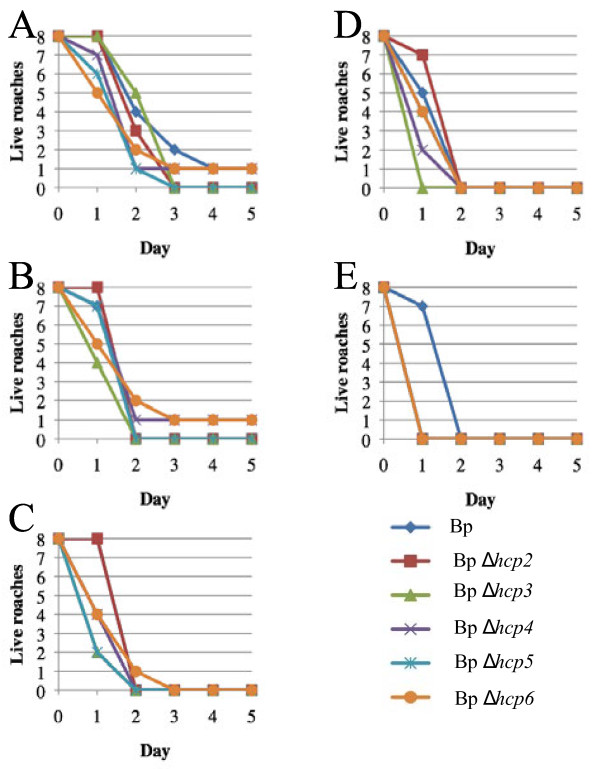
***B. pseudomallei*T6SS-2, T6SS-3, T6SS-4, T6SS-5, and T6SS-6 mutants are virulent in the MH cockroach.** (**A**) 10^1^ cfu. (**B**) 10^2^ cfu. (**C**) 10^3^ cfu. (**D**) 10^4^ cfu. (**E**) 10^5^ cfu. Bp, K96243; Bp *Δhcp2*, DDS0518A; Bp *Δhcp3*, DDS2098A; Bp *Δhcp4*, DDS0171A; Bp *Δhcp5*, DDS0099A; Bp *Δhcp6*, DDL3105A.

We next examined the relative virulence of the *B. pseudomallei Δhcp2, Δhcp3, Δhcp4, Δhcp5,* and *Δhcp6* mutants in MH cockroaches [9]. These mutants are each deficient in one of the other five T6SSs present in *B. pseudomallei* and all are virulent in the hamster (Table 1). Figure 3 shows that these strains are also virulent in the MH cockroach and all exhibit a clear dose response. The majority of MH cockroaches infected with a challenge dose of 10^1^ bacteria were dead by day 3 (Figure 3A), but most were dead by day 1 with a challenge dose of 10^5^ bacteria (Figure 3E). Interestingly, the LD_50_ results with these strains are remarkably similar in both MH cockroaches and hamsters (Table 1).

The virulence of two additional isolates of *B. pseudomallei* and two isolates of *Escherichia coli* were also tested in the MH cockroach. The LD_50_s of *B. pseudomallei* 1026b and MSHR305 were <10 bacteria and the LD_50_s for *E. coli* MC4100 and B/r were >10^5^ bacteria, the highest dose tested (Table 1). The results suggest that virulence for the MH cockroach is common among *B. pseudomallei* isolates and that not all gram-negative bacteria are pathogenic for this surrogate host (Table 1).

Taken together, the results demonstrate that *B. pseudomallei* is highly virulent in the MH cockroach and indicate that this insect might serve as a surrogate host for high throughput virulence screening assays. In addition, the MH cockroach challenge results are consistent with what is seen in the hamster model of infection and suggest that the primary function of the T6SS-1 is to evade the innate immune system.

### The MH cockroach can serve as a surrogate host for *B. mallei* and *B. thailandensis*

**Figure 4:**
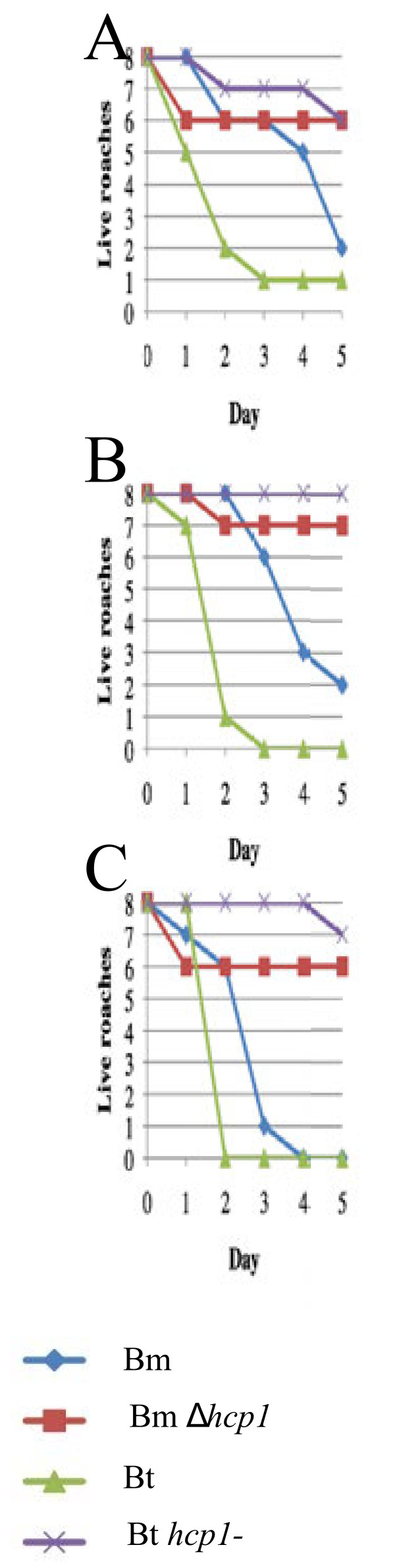
***B. mallei*and*B. thailandensis*are virulent for the MH cockroach and their T6SS-1 mutants are attenuated.** (**A**) 10^1^ cfu. (**B**) 10^2^ cfu. (**C**) 10^3^ cfu. Bm, SR1; Bm *Δhcp1*, DDA0742; Bt, DW503; Bt *Δhcp1*, DDII0868.

We also evaluated the virulence of *B. mallei* and *B. thailandensis* in the MH cockroach. The LD_50_s for *B. mallei* SR1 (Bm) and *B. thailandensis* DW503 (Bt) were < 10 bacteria (Table 1) and the number and rate of deaths increased as the challenge dose increased from 10^1^ to 10^3^ bacteria (Figure 4). Interestingly, *B. mallei* killed the MH cockroaches at a slower rate than *B. thailandensis* (and *B. pseudomallei*). It took only 2 days for *B. thailandensis* to kill 75% of the MH cockroaches with a dose of 10^1^ bacteria, whereas it took *B. mallei* 5 days (Figure 4A). Similar trends were apparent with challenge doses of 10^2^ and 10^3^ bacteria (Figure 4B and C). *B. mallei* does not kill rodents as quickly as *B. pseudomallei* and it is more fastidious than *B. pseudomallei* and *B. thailandensis*, so it may not be too surprising that it took longer to kill MH cockroaches [4]. These experiments demonstrate that *B. mallei* and *B. thailandensis* are both virulent in the MH cockroach and suggest that the MH cockroach might serve as a surrogate host for these bacterial species.

As mentioned above, *B. thailandensis* is considered to be avirulent in humans and exhibits a higher LD_50_ in mammalian models of infection than *B. mallei* and *B. pseudomallei*. Mammals, unlike MH cockroaches, possess both an innate and an acquired immune system. The fact that *B. thailandensis* is highly virulent in the MH cockroach may suggest that the acquired immune system plays an important role in defence against *B. thailandensis*. *B. mallei* and *B. pseudomallei*, on the other hand, may have developed mechanisms to subvert the acquired immune response in mammalian species.

T6SS-1 is a critical virulence determinant for *B. mallei* in the hamster model of infection [25] and for *B. thailandensis* in the C57BL/6 mouse model of infection [27]. We challenged MH cockroaches with *B. mallei* and *B. thailandensis hcp1* mutants and found that they were highly attenuated in this surrogate host (Table 1 and Figure 4). The LD_50_s for *B. mallei Δhcp1* and *B. thailandensis hcp1*^*-*^ were > 10^3^ bacteria on day 5, which was at least 100 times higher than their respective parental strains (Table 1 and Figure 4). The *B. mallei* results were indistinguishable from what was previously described for SR1 and *Δhcp1* using the hamster model of infection [25]. While the *B. thailandensis* strains used in this study have not been tested in hamsters, a *B. thailandensis* T6SS-1 mutant was recently shown to be avirulent in C57BL/6 mice by the aerosol route of infection [27]. Interestingly, MyD88^−/−^ mice were susceptible to the *B. thailandensis* T6SS-1 mutant, which suggests that T6SS-1 plays a role in evading the innate immune response [27]. The fact that *B. thailandensis hcp1*^-^ was attenuated in an insect host, which lacks an adaptive immune response, further supports the notion that the function of the T6SS-1 is to evade the eukaryotic innate immune system.

### *B. pseudomallei* replicates inside MH cockroach hemocytes

**Figure 5:**
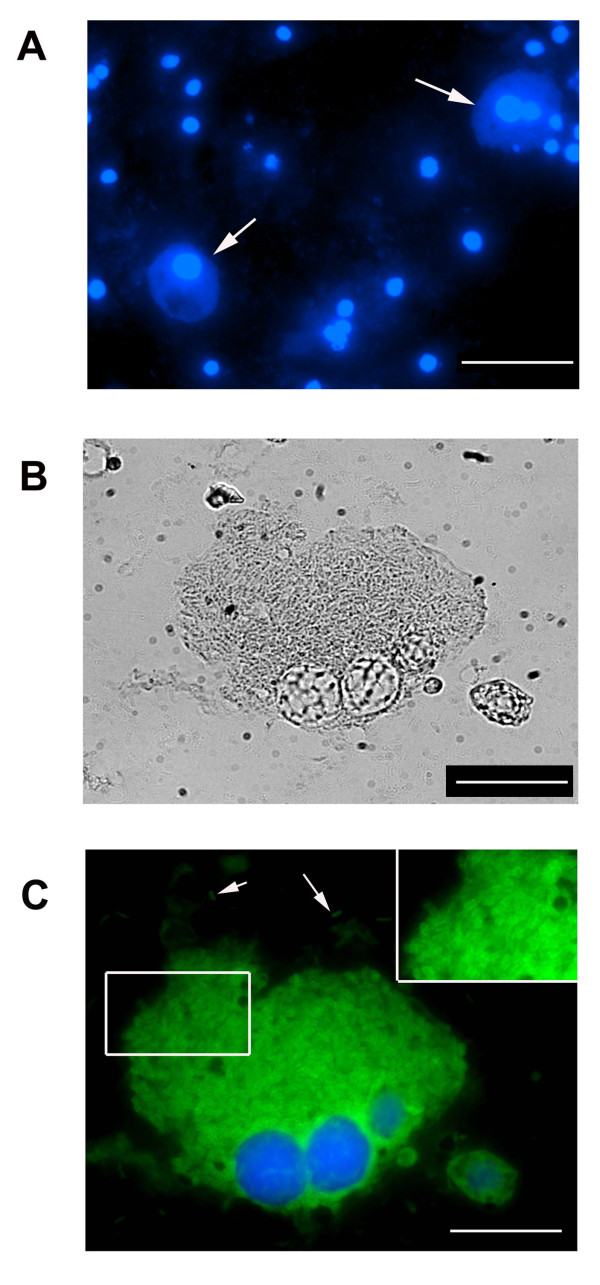
***B. pseudomallei*multiplies inside MH cockroach hemocytes.** Panel A is a representative micrograph of hemolymph obtained from a MH cockroach infected with *B. pseudomallei* K96243 and stained with DAPI. The white arrows show hemocytes that harbor intracellular *B. pseudomallei*. The white scale bar is 100 μm. Panels **B** and **C** show a higher magnification of a *B. pseudomallei*-infected hemocyte using bright field microscopy (**B**) and stained with DAPI and a *Burkholderia*-specific rabbit polyclonal antibody (**C**). The secondary antibody used, Alexa Fluor 588 goat anti-rabbit IgG, stained *B. pseudomallei* green. The magnified inset in **C** shows individual bacilli within the hemocyte cytosol and the white arrows show extracellular bacteria in the hemolymph. The white scale bars in **B** and **C** are 20 μm. The results are representative images from eight MH cockroaches infected with ~ 10^3^ cfu of *B. pseudomallei* K96243.

Hemocytes are a key component of the MH cockroach innate immune system and we next examined if *B. pseudomallei* might be exploiting these phagocytic cells to gain an upper hand in the host-pathogen interaction. A group of eight MH cockroaches were infected with ~ 10^3^*B. pseudomallei* K96243 and closely monitored for 48 h. MH cockroaches that appeared weak and lethargic were immediately separated from the others and hemolymph was obtained. The MH cockroach hemolymph, which contains phagocytic hemocytes, was fixed and stained with DAPI. Figure 5A shows a representative field containing the blue-staining nuclei from multiple hemocytes. As expected, the non-nuclear regions of most hemocytes could not be visualized with this fluorescent DNA stain. Interestingly, each field also contained one or two hemocytes in which the nucleus and the surrounding cytosol could be easily visualized (Figure 5A, white arrows). We speculated that these particular hematocytes might contain cytosolic *B. pseudomallei* and we stained the hemolymph with a polyclonal antibody that reacts with *B. pseudomallei*. Figure 5B and 5 C show a representative micrograph of a hematocyte engorged with cytosolic *B. pseudomallei*, suggesting that the bacteria are multiplying to high numbers inside these cells. Free bacteria can also be visualized in the hemolymph outside the hemocyte, but it is unclear if these cells are alive or dead (Figure 5B and 5 C). Some infected hemocytes appear to have multiple nuclei and may be multinucleated giant cells (MNGCs) (Figure 5). MNGC have been observed in cases of human melioidosis [28] and are often formed when *B.pseudomallei* infects murine macrophage-like cell lines in vitro [9]. The formation of *B. pseudomallei*-induced MNGCs in vivo in MH cockroaches is an exciting finding and indicates that MNGCs can form in non-adherent cells freely flowing within the hemolymph.

Based on these results, we hypothesize that *B. pseudomallei* is able to survive the innate immune system of the MH cockroach by establishing an intracellular niche within the hemocyte. Infected hemocytes harboring numerous cytosolic bacteria may fuse with uninfected hemocytes to form MNGCs, which may serve as a reservoir for continued bacterial replication and protection from the antimicrobial peptides present in the surrounding hemolymph. The amplification of bacteria within phagocytic hemocytes, and their subsequent release, may eventually overwhelm the MH cockroach and lead to death.

## Conclusions

Our findings indicate that the Madagascar hissing cockroach can serve as a surrogate host for the analysis of host-pathogen interactions with *B. pseudomallei*, *B. mallei*, and *B. thailandensis*. Using this system, we were able to detect virulence differences between parental strains and T6SS-1 mutants that were consistent with what was seen in rodent models of infection. *B. pseudomallei* K96243 demonstrated the ability to multiply inside insect hemocytes and form MNGCs, which may be the primary mechanism by which it avoids killing by the MH cockroach innate immune system. The MH cockroach will probably be useful for high throughput virulence screening assays with these *Burkholderia* species as well as other bacterial pathogens.

## Methods

### Bacterial strains, plasmids, and growth conditions

The bacterial strains and plasmids used in this study are described in Table 2. *E. coli*, *B. pseudomallei*, and *B. thailandensis* were grown at 37°C on Luria-Bertani (Lennox) agar (LB agar) or in LB broth. When appropriate, antibiotics were added at the following concentrations: 15 μg of gentamicin (Gm), 25 μg of streptomycin (Sm), and 25 μg of kanamycin (Km) per ml for *E. coli* and 25 μg of polymyxin B (Pm) and 25 μg of Gm per ml for *B. thailandensis*. *B. mallei* was grown at 37°C on LB agar with 4% glycerol or in LB broth with 4% glycerol. All bacterial strains were grown in broth for ~ 18 h with constant agitation at 250 revolutions per minute. Phosphate-buffered saline (PBS) was used to make serial dilutions of saturated bacterial cultures and the number of cfu present in the starting culture were determined by spreading 100 μl aliquots onto agar media and incubating for 24–48 h. A 20-mg/ml stock solution of the chromogenic indicator 5-bromo-4-chloro-3-indolyl-b-D-galactoside (X-Gal) was prepared in N,N-dimethylformamide, and 40 μl was spread onto the surface of plate medium for blue/white screening in *E. coli* TOP10. All manipulations with *B. pseudomallei* and *B. mallei* were carried out in class II and class III microbiological safety cabinets located in designated biosafety level 3 (BSL-3) laboratories.

**Table 2:** Strains and plasmids used in this study

**Strain or plasmid**	**Relevant characteristics^a^**	**Source or reference**
*E. coli*		
TOP10	General cloning and blue/white screening	Invitrogen
S17-1	Mobilizing strain with transfer genes of RP4 integrated on chromosome; Sm^r^, Pm^s^	[34]
MC4100	K-12 laboratory strain	[35]
B/r	B laboratory strain	[36]
*B. pseudomallei*		
K96243	Isolated in Thailand from a diabetic patient with a clinical history of short incubation, septicemic infection, and rapid progression to death	[37]
DDS1498A	K96243 derivative harboring a 162-bp in-frame deletion mutation in *hcp1* (*Δhcp1*)	[9]
DDS0518A	K96243 derivative harboring a 303-bp in-frame deletion mutation in *hcp2* (*Δhcp2*)	[9]
DDS2098A	K96243 derivative harboring a 186-bp in-frame deletion mutation in *hcp3* (*Δhcp3*)	[9]
DDS0171A	K96243 derivative harboring a 321-bp in-frame deletion mutation in *hcp4* (*Δhcp4*)	[9]
DDS0099A	K96243 derivative harboring a 192-bp in-frame deletion mutation in *hcp5* (*Δhcp5*)	[9]
DDL3105A	K96243 derivative harboring a 216-bp in-frame deletion mutation in *hcp6* (*Δhcp6*)	[9]
DDS1503-1A	K96243 derivative harboring a deletion of the 743-bp *Stu*I fragment at the 5’ end of *vgrG1* (*ΔvgrG1-5’*)	[9]
DDS1503-2A	K96243 derivative harboring a deletion of the 894-bp *Pst*I fragment at the 3’ end of *vgrG1* (*ΔvgrG1-3’*)	[9]
1026b	Isolated in Thailand from a human case of septicemic melioidosis with skin, soft tissue, and spleen involvement	[30]
MSHR305	Isolated from the brain of a fatal human melioidosis encephalomyelitis case in Australia	[38,39]
*B. mallei*		
SR1	ATCC 23344 sucrose-resistant derivative	[40]
DDA0742	SR1 derivative harboring a deletion of the 156 bp *Nar*I–*Sfu*I fragment internal to *hcp1*; *Δhcp1*	[25]
*B. thailandensis*		
DW503	E264 derivative; *Δ(amrR-oprA)* (Gm^s^) *rpsL* (Sm^r^)	[41]
DDII0868	DW503::pGSV3-0868; Gm^r^; *hcp1*^*-*^	This study
Plasmids		
pCR2.1-TOPO	3,931-bp TA vector; pMB1 *oriR*; Km^r^	Invitrogen
pCR2.1-0868	pCR2.1-TOPO containing 342-bp PCR product generated with II0868-up and II0868-dn	This study
pGSV3	Mobilizabile Gm^r^ suicide vector	[42]
pGSV3-0868	pGSV3 derivative containing *Eco*RI insert from pCR2.1-0868	This study

^a^ r, resistant; s, susceptible.

### PCR

The two deoxyribonucleotide primers used for PCR amplification of an internal gene fragment of *B. thailandensis* BTH_II0868 (*hcp1*) were purchased from Invitrogen (Frederick, MD) and designated II0868-up (5’-AGGGCAAGATTCTCGTCCAG-3’) and II0868-dn (5’-TCTCGTACGTGAACGATACG-3’). The PCR product was sized and isolated using agarose gel electrophoresis, cloned using the pCR2.1-TOPO TA Cloning Kit (Invitrogen), and transformed into chemically competent *E. coli* TOP10. PCR amplification was performed in a final reaction volume of 100 μl containing 1X Taq PCR Master Mix (Qiagen), 1 μM oligodeoxyribonucleotide primers, and approximately 200 ng of *B. thailandensis* DW503 genomic DNA. PCR cycling was performed using a PTC-150 MiniCycler with a Hot Bonnet accessory (MJ Research, Inc.) and heated to 97°C for 5 min. This was followed by 30 cycles of a three-temperature cycling protocol (97°C for 30 s, 55°C for 30 s, and 72°C for 1 min) and one cycle at 72°C for 10 min.

### DNA manipulation and plasmid conjugation

Restriction enzymes, Antarctic phosphatase, and T4 DNA ligase were purchased from Roche Molecular Biochemicals and were used according to the manufacturer’s instructions. DNA fragments used in cloning procedures were excised from agarose gels and purified with a GeneClean III kit (Q · BIOgene). Bacterial genomic DNA was prepared by a previously described protocol [29]. Plasmids were purified from overnight cultures by using Wizard Plus SV Minipreps (Promega). Plasmid pGSV3-0868 (Table 2) was electroporated into *E. coli* S17-1 (12.25 kV/cm) and conjugated with *B. thailandensis* for 8 h, as described elsewhere [30]. Pm was used to counterselect *E. coli* S17-1 (pGSV3-0868).

### MH cockroach housing and manipulation

Madagascar hissing cockroaches, *Gromphadorhina laevigata*, were purchased from Carolina Biological Supply Company (Burlington, NC) as 1–2 inch nymphs (Figure 1) and were housed in the dark at room temperature in a 7.5" w x 11.75 l x 5" h mouse cage with a filtered top (Allentown Caging Equipment Co., Inc., Allentown, NJ). The bottom of the cage was lined with cocoa mulch and a thin layer of petroleum jelly was spread around the top portion of the cage to prevent MH cockroaches from climbing up the sides. Dog food was spread on the bottom of the cage for food and the top of a petri dish was inverted and filled with water for drinking. On occasion, sliced apple wedges were placed in the cage as an additional source of food.

For bacterial infection experiments, 1.5-2 inch juvenile MH cockroaches were used (Figure 1). We also tested larger MH cockroaches (> 3 inches) and they displayed the same susceptibility as the juveniles (data not shown). Bacteria were inoculated into the dorsal abdominal section of MH cockroaches, between the third and the fifth terga (from the posterior), using a 1 ml syringe fitted with a 3/8 inch, 26-gauge needle (see Figure 1). The syringe was loaded into a Tridak STEPPER series repetitive pipette (Tridak LLC, Torrington, CT) and a 25 μl aliquot was injected into MH cockroaches. A group of eight infected MH cockroaches were placed in a 16-ounce plastic container with a few pieces of dog food and 1–2 ml of water. The containers were placed in a 37°C incubator and deaths were recorded for 5 days. Food and water levels were checked daily and replenished if needed. The LD_50_s were calculated 5 days postinfection according to the Reed-Muench method [31].

### Extraction and staining of hemolymph from infected MH cockroaches

Eight MH cockroaches were infected with ~ 10^3^*B. pseudomallei* K96243 and monitored daily as described above. Hemolymph was extracted from MH cockroaches that were lethargic and on the verge of death. Holding the MH cockroach with its ventral side up, one hind leg was folded up towards the head to expose the membrane at the base of the leg. The membrane was punctured with a 26-gauge needle and hemolymph was immediately collected using a P200 Gilson PIPETMAN. We used a pipette tip cut with scissors approximately a 1/2 inch from the end to aid in uptake of the viscous hemolyph. The amber-colored hemolymph was transferred to a glass slide, allowed to air dry, and then fixed with methanol. The samples were initially stained with 4′, 6-diamidino-2-phenylindole (DAPI) and viewed on a Nikon Eclipse TE2000-S inverted microscope equipped with a Spot-RT digital camera (Image Systems, Columbia, MD). Subsequently, the samples were incubated for 1 h with a 1:1000 dilution of rabbit polyclonal *Burkholderia* antiserum [32] and then reacted for 1 h with a 1:500 dilution of an Alexa Fluor 588 goat anti-rabbit IgG secondary antibody (Molecular Probes) and visualized by fluorescence microscopy.

## Abbreviations

MH, Cockroach; Madagascar, Hissing cockroach; T6SS-1, Ccluster 1 type VI secretion system; LD50, 50% lethal dose; MNGC, Multinucleated giant cell; Cfu, Colony forming units; DAPI, 4′, 6-diamidino-2-phenylindole.

## Authors’ contributions

NAF conceived use of the MH cockroach as a surrogate host, contributed to the experimental design, and helped draft the manuscript. WJR was involved with the extraction, staining, and fluorescence microscopy of MH cockroach hemolymph. WA participated in the study design and conducted experiments. DD designed and conducted the experiments and drafted the manuscript. All authors read and approved the final manuscript.

## Authors’ information

Opinions, interpretations, conclusions, and recommendations are those of the author and are not necessarily endorsed by the U.S. Army or Department of Homeland Security.
